# gLeiden: accelerated community detection algorithms using directed and undirected graphs on GPUs

**DOI:** 10.1093/bioadv/vbaf327

**Published:** 2026-01-27

**Authors:** Beenish Gul, Maria Murach, Stefan Bekiranov, Kevin Skadron

**Affiliations:** Department of Computer Science, University of Virginia, Charlottesville, Virginia, 22903, United States; Department of Genome Sciences, University of Virginia School of Medicine, Charlottesville, Virginia, 22903, United States; Department of Biochemistry and Molecular Genetics, University of Virginia School of Medicine, VA 22908, Charlottesville, United States; Beirne B. Carter Center for Immunology Research, University of Virginia School of Medicine, VA 22908, Charlottesville, United States; Immunology, Imaging, and Informatics in Precision Immunomedicine (iPRIME) Program, University of Virginia School of Medicine, VA 22908, Charlottesville, United States; Department of Computer Science, University of Virginia, Charlottesville, Virginia, 22903, United States

## Abstract

**Motivation:**

Community detection methods are applied to single cell RNA sequencing (i.e. scRNA-seq) and mass cytometry data to efficiently identify major cell types and their subtypes, but their computational demands increase, particularly given the substantial growth in dataset sizes. The Leiden algorithm, an emerging method in this field, offers inherent parallelism that remains underutilized due to the limited parallel processing capabilities offered by today’s modern multi-core CPUs, which have fewer than 100 cores (typically 32–64 CPUs). However, Leiden can achieve significant performance gains when implemented on GPUs. GPUs offer high memory bandwidth and an extensive array of parallel processing units that map well to the parallelism in Leiden. As far as we know, cuGraph is the only implementation that has mapped the Leiden algorithm to GPUs, using a blend of Python and C languages. However, it only supports undirected graphs, potentially discarding the valuable information carried by edge directionality. In addition, this Python implementation for GPUs is comparatively slower than a C/C++ based implementation, reducing the significant performance gains provided by a GPU-based speedup. Conversely, a C/C++ based implementation optimizes performance more effectively, ensuring an accurate baseline comparison when performing GPU acceleration.

**Results:**

We developed a tool named gLeiden, a lightweight CUDA C++ based GPU implementation of the Leiden algorithm and, to the best of our knowledge, the very first GPU implementation that supports directed graphs, which generally demands nearly twice the computational time and memory resources compared to undirected graphs. The results show that our directed gLeiden outperforms the directed cLeiden version and shows 11× and 12× speedup on very large datasets. Our undirected ucLeiden and ugLeiden implementations significantly outperform the original Java version, with up to 42× speedup on large datasets. However, when comparing the undirected ugLeiden version with cuGraph, ugLeiden performance is comparable on smaller datasets and 58% faster on larger datasets. These results position our GPU-based Leiden implementation as a high-performance alternative to existing state-of-the-art community detection tools.

**Availability and implementation:**

The source code and sample data are available at: https://github.com/Beenishgul/Leiden and https://figshare.com/s/3b51e463a56e2a374bdf

## 1 Introduction

Graph-based clustering or community detection is at the heart of current single cell data analysis pipelines (El Mouden *et al.* 2023). Community detection is used to identify major cell types and their subtypes from single cell data (Zhang *et al.* 2023). Recent advancements in genomics have introduced community detection methods that enhance clustering accuracy for functional genomic data compared to traditional heuristic approaches. Unlike prior methods, community detection algorithms focus on maximizing modularity, which measures the strength of connections within clusters, leading to better identification of tightly connected groups. The modularity function measures the strength of communities (Hairol Anuar *et al.* 2021), as the relationship between the links within the communities and those between communities. Higher modularity corresponds to densely connected communities and better quality (Hairol Anuar *et al.* 2021). One of the most recently introduced community detection algorithms is the Leiden algorithm (Traag *et al.* 2019), an improvement to Louvain (Waltman and van Eck 2013). Leiden is gaining popularity in functional genomics for clustering, due to its superior result quality compared to the Louvain algorithm. The key improvement is Leiden’s refine-partition step, which addresses weakly connected communities that Louvain might miss. This step revisits newly formed communities, identifying and isolating nodes whose removal would disconnect others. Thus, disconnected nodes are reassigned to their original groups, effectively becoming individual communities.

The process begins with an initial random predefined partitioning of nodes into communities (each node in its community). It evaluates the modularity gain by considering each node’s potential relocation to neighboring communities and comparing resulting modularity changes. It then moves the node to the community with the highest increase in modularity or retains its current community if no relocation improves modularity. This multi-phase refinement continues until no further modularity improvement is possible. While more accurate than Louvain, Leiden is computationally intensive, and traditional CPUs, even multi-core ones, cannot fully exploit its parallelism, making execution time costly. However, the algorithm scales well with thousands of cores and shows significant performance improvements on parallel computing platforms such as GPUs, which offer much greater computational power than CPUs (Li *et al.* 2016, Fatima *et al.* 2025) which have fewer than 100 cores, typically 32–64. Leveraging the power of GPUs can significantly accelerate these computations by exploiting their massively parallel architecture, allowing for faster and more efficient algorithm execution. Along with speeding up one clustering instance, this would also enable further applications, including identifying the optimal number of communities or clusters (Liu *et al.* 2021) and ensemble clustering (Wang *et al.* 2024, Goggin and Zunder 2024), which require multiple clustering runs that can be prohibitively time-consuming for datasets containing millions to tens of millions of cells.

Moreover, we are unaware of any work that maps Leiden to GPUs using the C/C++ language and supports directed graphs. However, direction is vital for community detection in networks such as Bayesian networks, because it represents causal relationships rather than mere correlations (Rottman and Hastie 2014, Shen *et al.* 2024). Indeed, directed graphs play a central role in representing causal molecular networks in cells (Huber *et al.* 2007). For example, in gene regulatory networks, direction shows how one gene’s expression regulates another, which is essential for understanding biological processes and designing interventions (Levine and Davidson 2005, Huber *et al.* 2007, Chai et al. 2014, Wei *et al.* 2024). To our knowledge, there is a mixed Python/C undirected implementation of Leiden on GPU (Kang *et al.* 2023), which relies on various Python libraries and dependencies, which could complicate the process. Python’s interpreted nature introduces overhead, affecting the overall performance for computationally intensive tasks such as Leiden compared to natively compiled languages such as C/C++. To address the challenges outlined above, we present an optimized C++ language-based Leiden implementation and then map it to the GPU. To the best of our knowledge, this implementation is the first that supports directed graphs.

**Extending Leiden to directed graphs: key challenges:** Extending Leiden to directed graphs and generalizing loss functions is non-trivial due to both algorithmic and representational challenges. In undirected networks, symmetric edge weights $w_{ij}=w_{ji}$ allow a single adjacency structure and simplify gain computation. Directed networks break this symmetry, requiring separate incoming and out-going edge handling and ΔQ computations (for both directions) as presented in Equation (1). Directed implementation has been avoided due to high time and space costs: flipping edges requires multiple additional arrays, tripling memory usage, and sorting these arrays incurs *O*(*n* log *n*) overhead. We introduce the simplified approach, which uses offset-based CSR transposition that deterministically places each out-neighbor into its correct in-neighbor position, eliminating expensive sorting and reducing memory movement.


(1) $$\Delta Q=\left[\frac{\sum in+2k_{in}}{m}-{\left(\frac{\sum t\text{ot}+k_{i}}{m}\right)}^{2}\right]-\left[\frac{\sum in}{m}-{\left(\frac{\sum t\text{ot}}{m}\right)}^{2}-{\left(\frac{k_{i}}{m}\right)}^{2}\right]$$

where $k_{i,\text{out}\to C}$ and $k_{i,\text{in}\to C}$ denote the sum of outgoing and incoming edge weights from node *i* to community *C*, respectively.

## 2 Methodology

The GPU architecture is composed of a scalable array of Streaming Multiprocessors (SMs). Modern GPUs, for example, NVIDIA’s Ampere architecture, consists of 6912 CUDA cores (NVIDIA) sliced into 108 Streaming Processors. To parallelize Leiden on GPUs, all graph data arrays, including neighbors, community assignments, in-degree, out-degree, and self-loops are transferred to the GPU via cudaMemcpy(), after which CUDA threads process nodes in parallel and evaluate their potential community moves.

The algorithm is structured around two main loops: an outer loop, which is dedicated to node handling, and an inner loop, which iterates through their respective neighbors. The outer loop is parallelized in such a way that each thread calculates the modularity gain by moving the assigned node to neighboring communities, providing the best modularity gain. The unique global thread index, known as globalThreadIdx in CUDA, ensures that each thread operates on a distinct subset of the data (node and its neighbors), with the inner loop’s execution managed by the corresponding thread in the outer loop.

Our findings indicate that parallelizing both the inner and outer loops concurrently introduce substantial synchronization challenges due to the varying number of neighbors per node. Nodes with fewer neighbors complete their computations faster than those with more, necessitating additional synchronization points. Consequently, threads processing nodes with fewer neighbors must frequently wait for those handling nodes with more neighbors. Moreover, excessive synchronization for updating values across threads can significantly degrade performance, as many threads repeatedly stall to maintain consistency. (Bradley 2012, Dalmia *et al.* 2022). To minimize the synchronization overhead, we only parallelize the outer loop in such a way that each thread takes the responsibility of calculating the modularity gain for one node with its neighbors and updates the corresponding information for its respective assigned node. In addition, the Leiden algorithm naturally reduces the graph size with each pass, simplifying subsequent passes. This natural reduction eliminates the need for nested parallelism as for the next iterations, neighbor’s list becomes smaller and faster.

In the refine-partition phase, each CUDA thread reassesses the modularity gain of nodes within the supernodes and merges two nodes under strict conditions: (i) both nodes must belong to the same supernode, and (ii) their merger must result in an increased modularity gain. The refinement phase is more efficient than the initial phase as it eliminates the need to traverse all nodes within the supernodes repeatedly. Instead, it tracks nodes that remain candidates for merging. In subsequent iterations, only unmerged nodes are considered for potential movement, substantially reducing computation overhead.

All phases run in parallel and are repeatedly applied to successive graphs until the graph structure completely converges.

## 3 Results

This section presents the performance analysis of the Leiden algorithm conducted on small, medium, and large graphs. Table 1 presents the datasets along with their input sizes and graph types, chosen to evaluate the application performance on CPU and GPU.

**Table 1:** Datasets for evaluations on CPU and GPU.

Graph type	Nodes	Edges
Single-cell mass spectrometry (CyTOF)	1.68M	25.2M
Single-cell mass spectrometry (CyTOF)	13M	195M
Single-cell mass spectrometry (CyTOF)	20M	300M

### 3.1 Testing environment and results

We evaluated both CPU- and GPU-based versions of the Leiden algorithm on a high-performance system, with specifications summarized in Table 2.

**Table 2:** System environment for CPU and GPU evaluations.

	CPU (host)	GPU (device)
Model	AMD EPYC 7742	NVIDIA A100
Cores/SMs	128	108
Threads/CUDA cores	256	6912
Clock speed	2.25 GHz	1.58 GHz
Memory	256 GB	40 GB
Data rate	204.8 GB/s × 2	1555 GB/s
TDP	225 W × 2	400 W

### 3.2 Experimental analysis

This section presents the performance analysis of the Leiden algorithm conducted on the datasets listed in Table 1. To benchmark the performance of our GPU-based Leiden (gLeiden) implementation, we compare it against three representative and widely-used baselines: (i) the original Java implementation of the Leiden algorithm, recognized as the canonical reference for the method; (ii) cuGraph’s GPU-accelerated community detection module, which implements a variant of Leiden optimized for NVIDIA architectures and is part of the RAPIDS AI ecosystem; and (iii) a CPU-only version of our own C++ Leiden (cLeiden) implementation. These baselines span both CPU- and GPU-based environments and vary in implementation language and optimization strategies, offering a representative spectrum of state-of-the-art community detection tools. Notably, both our GPU implementation and cuGraph leverage GPU parallelism, while the Java and C++ CPU versions provide strong baselines for single-threaded CPU performance. This comparative analysis highlights the effectiveness and efficiency of our approach across different hardware and software paradigms. The results of our directed cLeiden with directed gLeiden and the results of our undirected version with existing undirected Java/CPU and undirected cuGraph implementations.

**Figure 1:**
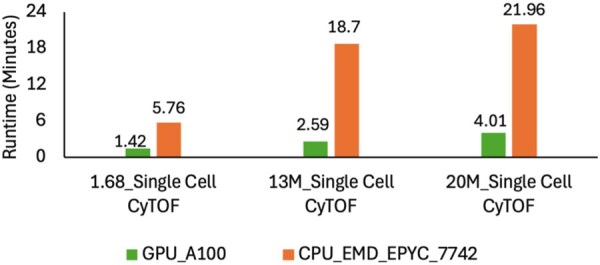
Runtime performance comparison of gLeiden versus cLeiden on large-scale directed graphs.

Figure 1 shows the execution time breakdown for all the datasets in Table 1, comparing both cLeiden and gLeiden. The results demonstrate a significant speedup achieved by the GPU implementation over the CPU implementation. We evaluated the performance on 256 threads per block configuration. From left to right, for the smaller dataset (first bar in Fig. 1), CPU execution is already extremely fast, the GPU, being even faster, produces an execution time so small that its bar is barely visible in comparison. We see a clear difference as the input size grows. On medium and larger datasets, the GPU version shows 8× and 6× speedup on large datasets compared to the CPU implementation, respectively.

The baseline Java/CPU and GPU/Python implementations are designed for undirected graphs, while the memory and time requirements for the directed version are nearly double. To ensure a fair performance comparison under similar time and memory constraints, we also implemented an undirected version of the Leiden algorithm. The CPU code was compiled with g++ using -O3 optimization which offers inlining, loop unrolling, and auto-vectorization (where applicable). We have also used the optimized STL libraries for sorting and reduce operations during initialization and intermediate preprocessing for aggregate phase. We compare the performance of our undirected C++/CPU (ucLeiden) and C++/GPU (ugLeiden) implementations with those of Java and cuGraph. ucLeiden outperforms the original Java version, achieving speedups of 12× and 8.5×, respectively. Additionally, our undirected C++/GPU implementation is 38× and 42× faster than Java on very large datasets. When comparing the undirected GPU version with cuGraph, performance is comparable on smaller datasets, but cuGraph is 58% slower on larger datasets.

**Figure 2:**
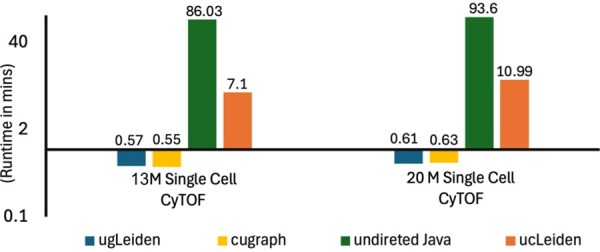
Comparison of ugLeiden with three alternatives: (i) undirected Java, (ii) undirected cuGraph, and (iii) ucLeiden. These baselines were selected to reflect common choices across GPU, CPU, and Java-based environments.

These results demonstrate an improvement over cuGraph, primarily due to the use of memory reuse techniques. Figure 2 presents the detailed analysis performed on undirected versions of the Leiden algorithm. In Fig. 2, the first two bars representing our undirected implementation and cuGraph are not visible (above the bar) because they are significantly faster than the Java and C++ implementations. We also reduce the number of nested loops and avoid costly search or find operations when creating new partitions and neighbors. Instead, we reuse data directly by linking the relocated nodes to the new and communities (where they have moved) with new pointers. This eliminates redundant computations and reduces the execution time compared to cuGraph.

**Figure 3:**
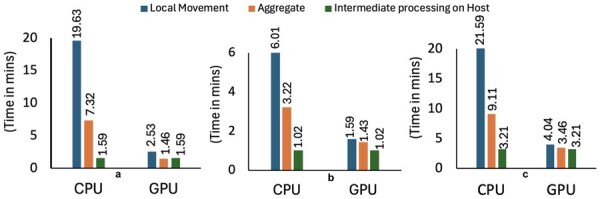
Breakdown of the runtime for the three phases of the Leiden algorithm on CPU and GPU, reporting host and device times before and after parallelization for datasets with (a) 1.68M, (b) 13M, and (c) 20M cells.

Figure 3 illustrates the runtime breakdown of all three phases, emphasizing the phase-wise performance improvements achieved by the GPU implementation cover the CPU.

We evaluated our experiments on the AMD EPYC 7742, CPU performance remains far slower than the GPU (Fig. 1). Leiden is heavily memory-bound, and the EPYC’s 410 GB/s bandwidth is much lower than the 1.5 TB/s of modern GPUs. GPUs also achieve higher throughput by running thousands of threads and hiding memory latency, an ability CPUs cannot match even with many cores.

In addition to runtime scalability, practical deployment of gLeiden depends on its GPU memory footprint. We therefore measured the VRAM usage across datasets. For dataset with 13M nodes and ≈195M edges, gLeiden required ≈19 GB of GPU memory while for or a dataset with 20M nodes and ≈300M edges, we measure the memory usage to be ≈30 GB (geomean). Memory consumption scales approximately linearly with the number of node and edges, O∣V∣+∣E∣. These results indicate that gLeiden can be executed on commonly available GPUs with ≥24 GB of VRAM for graphs up to ≈13M nodes and scales further on higher-end GPUs (e.g. A100 40 GB or 80 GB). We measured GPU memory usage (VRAM) by recording the free GPU memory before and after loading the graph into device memory using the CUDA runtime API cudaMemGetInfo.

To further evaluate the reliability of gLeiden under these conditions, we also assessed stability by repeating each experiment 10× with different random seeds. As expected, Leiden produces slightly different community assignments across runs due to its non-deterministic nature. However, the overall community structure remains consistent, with a mean NMI of 0.96 and moderate runtime variation (≥3% standard deviation), indicating that gLeiden provides both scalable and stable results across multiple runs.

### 3.3 Ground truth verification

The LFR benchmark is a widely used approach for evaluating community detection algorithms (Lancichinetti *et al.* 2008). It generates graphs that mimic real-world complex networks, and provides the network structure with planted communities. The performance of the algorithm is evaluated on the basis of how accurately the detected communities match the ground truth provided by the generator, particularly as the task becomes more challenging with increasing mixing factor/resolution factor μ. While some real-world networks, such as interest-based groups in online social networks from the SNAP collection, come with assumed ground truth communities, we consider synthetic ground truth to be more reliable for our analysis.

**Figure 4:**
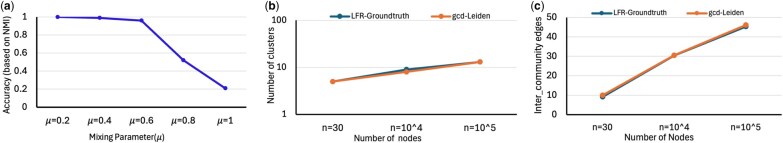
(a) LFR benchmark ($n=10^{5}$): effect of inter-community edges on ground truth detection accuracy. (b) Number of remaining clusters identified using the ground truth. (c) Inter-community edges identified using the ground truth on directed graphs.

Figure 4 illustrates the agreement between the detected and ground truth communities using the graph-structural rand index (where 1 indicates perfect agreement). The results show that the approach effectively matches the communities detected by ground truth even under significant noise (μ = 0.8). To assess cluster similarity, we compared the number of communities produced by our implementation against the LFR benchmark on the same generated graph. For each community, we also verified that inter-community edge-weight sums matched the LFR ground truth.

**Figure 5:**
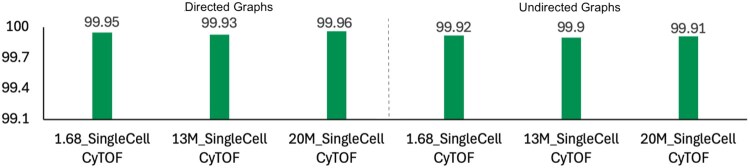
Comparison of clustering quality between CPU and GPU implementations on directed and undirected graphs.

We also compare the results by using vertex-to-community mapping to identify and analyze corrections of community assignments at the node level. Figure 5 presents a comparison for both directed and undirected graphs. In part (a), we compare gLeiden and cLeiden, while in part (b), we compare ucLeiden with ugLeiden. For each node pair, community assignments are compared across the graphs listed in Table 1, with accuracy shown on the y-axis as a percentage. While the mappings are largely consistent, a small number of differences in community assignments may occur due to tie-breaking situations and the inherent randomness in the Leiden (and Louvain) algorithms. For example, a node may have equal modularity gain from multiple neighboring communities, or the gain may be zero, leading to different outcomes depending on tie-breaking rules or iteration order.

## 4 Conclusions and future work

In this paper, we demonstrate that the Leiden algorithm excels at identifying well-connected communities but is complex and time-consuming, particularly when executed on multicore CPUs. However, GPUs are well-suited for this task, offering a promising solution for speeding up the computation. Importantly, while achieving this speedup, maintaining the quality of clustering remains a critical consideration. We also show that studies including (Levine and Davidson 2005, Chai et al. 2014, Rottman and Hastie 2014, Shen *et al.* 2024) are proof that consideration of edge directionality is important while performing community detection. Our evaluations show that GPU accelerated versions of the Leiden algorithm are clear winners when compared to the CPU versions.

Future work includes integrating our GPU implementation with Processing-In-Memory (PIM) technology to accelerate the partitioning phase of the Leiden algorithm, optimizing data handling and computational efficiency.
