# Correction to: gLeiden: accelerated community detection algorithms using directed and undirected graphs on GPUs

**DOI:** 10.1093/bioadv/vbag177

**Published:** 2026-07-09

This is a **correction** to:

Beenish Gul, Maria Murach, Stefan Bekiranov, Kevin Skadron, gLeiden: accelerated community detection algorithms using directed and undirected graphs on GPUs, Bioinformatics Advances, Volume 6, Issue 1, 2026, vbaf327, https://doi.org/10.1093/bioadv/vbaf327

In the originally published version of this manuscript, author, Stefan Bekiranov’s, surname was misspelled.

This error has been corrected.

